# Shear bond strength of metallic brackets bonded to enamel pretreated with Er,Cr:YSGG LASER and CPP-ACP

**DOI:** 10.1186/s12903-021-01669-y

**Published:** 2021-06-14

**Authors:** Yomna A. Nabawy, Tarek N. Yousry, Nadia M. El-Harouni

**Affiliations:** Department of Orthodontics, Faculty of Dentistry, Alexandria University, Champollion St., Azarita, P. O. Box 21521, Alexandria, Egypt

**Keywords:** Laser, Er,Cr:YSGG, Prevention, CPP-ACP, Pre-treatment, SBS, Failure mode

## Abstract

**Background:**

Increased risk of enamel demineralization during and after orthodontic treatment raises the demand for better preventive measures including combinations of laser, CPP-ACP, and fluoride. The combination of Er,Cr:YSGG laser with CPP-ACP was proved to have a synergetic effect compared to each of them alone. Shear bond strength (SBS) of orthodontic brackets bonded to the enamel surface after being treated with preventive measures is critical. The aim of this study was to compare the SBS and failure mode of metallic brackets bonded to teeth with no pretreatment and pretreated enamel surface, either with Er,Cr:YSGG laser alone or combined with CPP-ACP.

**Methods:**

Sixty sound extracted human premolar teeth were allocated randomly to 3 groups: In Group 1 (control), teeth were etched and bonded directly; in Group 2, laser pretreatment of the enamel surface was done followed by etching and bonding as in the control group; in Group 3, the enamel surface was lased then CPP-ACP was applied according to the manufacturer instructions, etched and bonded. SBS and Adhesive remnant index (ARI) were evaluated.

**Results:**

No significant differences were found between the 3 groups neither in the SBS nor in the ARI scores.

**Conclusions:**

The use of combined Er,Cr:YSGG laser with CPP-ACP as a preventive measure before bonding orthodontic brackets does not endanger the bracket’s bonding strength.

## Background

Patients seeking orthodontic treatment are always at risk of developing white spot lesions (WSLs) and subsequently cavitation and caries around orthodontic brackets, particularly in poor oral hygiene patients [1]. The prevalence of WSLs ranges between 2 and 96% [2–5] and could appear after only one month of orthodontic treatment [1, 6].

The treatment of WSLs can be difficult after the removal of fixed appliances, and they rarely disappear completely. WSLs influence esthetics, resulting in patients’ dissatisfaction with their final smile [7]. This raises the demand for greater attention in providing better preventive measures and decreasing the amount of enamel demineralization.

Good oral hygiene and lower carbohydrate consumption are important ways to preserve enamel during orthodontic treatment [7, 8]. Nonetheless, the presence of bonded attachments reduces efficient oral hygiene maintenance [2]. In patients with a high risk of developing WSLs during orthodontic therapy, additional preventive measures are required, [9] including treatment of enamel surface with different chemicals such as fluoride products [10–12] and Casein phosphopeptide amorphous calcium phosphate (CPP-ACP) [13].

Several studies showed that sub-ablative laser irradiation increases the acid resistance of enamel [14–17], though the actual mechanism remains unclear [14, 18]. Different types of laser irradiation could provide an effective strategy to reduce enamel demineralization, including CO_2_, neodymium-doped yttrium aluminum garnet (Nd:YAG), erbium-doped yttrium aluminum garnet (Er:YAG), erbium, chromium: yttrium-scandium-gallium-garnet (Er,Cr:YSGG), diode and argon lasers [19].

Several attempts have been carried out to increase the effectiveness of the prevention, by combining different measures to provide better results. Previous studies evaluated the combination of CPP-ACP with fluoride (CPP-ACPF) [20], laser with fluoride [21], laser with CPP-ACP [22] as well as laser with CPP-ACPF [23], of which several demonstrated an increased or synergetic preventive potential when laser was combined with other preventive measures [20, 21, 23].

Adel et al. [24] in 2020 compared Er,Cr:YSGG, CPP–ACP and their combination in terms of WSLs prevention and concluded that the combined use of Er,Cr:YSGG with CPP–ACP resulted in a significantly higher preventive potential than using each of them alone.

Despite their potential ability to prevent and re-mineralize WSLs, pretreatment of the enamel surface with such prophylactic measures may affect the bond strength of orthodontic brackets. Previous in vitro researches were conducted to evaluate the shear bond strength (SBS) after pretreatment of the enamel surface with different preventive measures. The results revealed either a significant increase [25, 26] or a significant decrease [27] or no significant difference in the SBS [15, 28]. However, a lack of studies evaluating the SBS of orthodontic brackets bonded to the enamel surface after being treated by sub-ablative Er,Cr:YSGG laser alone or when combined with CPP-ACP was observed.

## Methods

This randomized controlled in-vitro study was conducted to compare the shear bond strength (SBS) and adhesive remnant index (ARI) of orthodontic brackets bonded to enamel surface with no pretreatment (control), sub-ablative Er,Cr:YSGG laser pre-treatment, and combination of sub-ablative Er,Cr:YSGG laser and CPP-ACP pre-treatment.

The research was approved by the institutional review board at the Faculty of Dentistry, Alexandria University (IRB:00010556–IORG:0008839). All the methods were carried out in accordance with CRIS guidelines and regulations. The entire study was conducted at Orthodontic Departments in Alexandria University and Biomaterial Department in Ein Shams University.

### Sample grouping and preparation

Sample size estimation was calculated using Power and Sample Size Calculation computer software (Epi-Info 7 software, Atlanta, GA, USA). At α = 0.05 and a power of 0.95, a total of 60 premolars was needed [29].

Sixty sound human premolar teeth freshly extracted for orthodontic needs were collected. An informed consent was signed by each subject to allow the use of the premolars. A legal guardian signed the consent if the subject was under 18 years of age. The teeth had to show no cracks nor decalcification to be included in the current study. Teeth were cleaned under tap water, pumiced, then stored in saline (0.9% NaCl) solution which was changed weekly. Upon starting the experiment each tooth was assigned a number from 1 to 60 for identification purpose and was stored in a separate labeled container filled with artificial saliva *(*20 mmol/l NaHCO_3_, 3 mmol/l NaH_2_PO_4_, 1 mmol/l CaCl_2_, at neutral pH 7*)* [30] which was changed daily. Using a random number generator, teeth were divided into three experimental groups.

In group 1 (control), the teeth did not receive any pre-treatment before bonding. 37% phosphoric acid gel was applied (Meta Etchant, Meta Biomed, Korea) for 30 s, rinsed off for a minimum of 5 s, and the teeth were air-dried with oil free air until a chalky white appearance was noticed. A thin layer of light cured bond (Ortho Solo Universal Sealant and Bond Enhancer, Ormco Corp. Glendora, California, USA) was applied with a micro-brush and air-dried with oil free air. After applying the Grengloo adhesive (Ormco,. Glendora, California, USA) to the brackets (Ormco Mini 2000. Ormco Corp. Glendora, California, USA). They were adjusted to the center of the buccal surface, pressed firmly and the excess was removed. Each of the mesial and distal surfaces was cured for 20 s. (Woodpecker i-led, 2300 mW/cm, woodpecker, china). The bonding procedure was done by one operator following the manufacturer’s instructions.

In group II (Er,Cr:YSGG pre-treatment), sub-ablative Er,Cr:YSGG laser (WaterLase iPlusTM, Biolase Inc., USA) was adjusted to a 2.78 μm wavelength, 0.25 W power, 12.5 mJ pulse energy, 8.5 J/cm^2^ energy density, 20 Hz frequency and 140 μs pulse duration [24]. An MZ6 tip was inserted into the gold handpiece, held 1 mm away, and perpendicular to the enamel surface. An endodontic file was fixed at the gold handpiece head to guarantee this distance, providing the same spot size irradiation to each tooth [31]. Irradiation was done slowly with a uniform speed of 2 mm/sec in a scanning style once in a horizontal then in a vertical direction with 11% air and no water-cooling system for 20 s [24]. Visual inspection of the enamel surface was done ensuring no surface morphology changes, then bonding was done with the same previously mentioned protocol.

In group III (Er,Cr:YSGG and CPP-ACP pre-treatment), after laser irradiation, 10% w/v CPP-ACP Tooth Mousse® (GC Corporation, Tokyo, Japan) was applied on the whole buccal surface for 5 min then rinsed. This was repeated for 5 successive days as recommended by the manufacturer and the same bonding procedure was done.

To simulate approximately 1 year in the oral environment, all the specimens were subjected to thermocycling (SD Mechatronik, Feldkirchen-Westerham, Germany). 1,000 thermocycles were done in water between 5 °C and 55 °C with a dwell time of 30 s and a transfer time of 5 s [32].

**Fig. 1:**
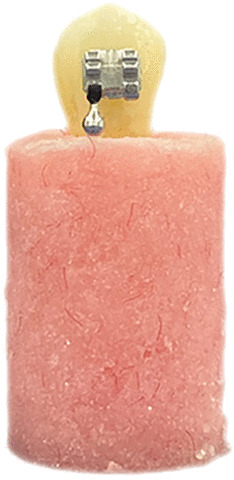
Tooth embedded in a chemical cure acrylic resin cylindrical mold

Roots of all teeth were embedded in a chemical cure acrylic resin cylindrical mold (Fig. 1). A surveyor was used, ensuring that the buccal surface of each tooth was perpendicular to the bottom of the mold and the molds were stored in the same container again.

### Shear bond strength

**Fig. 2:**
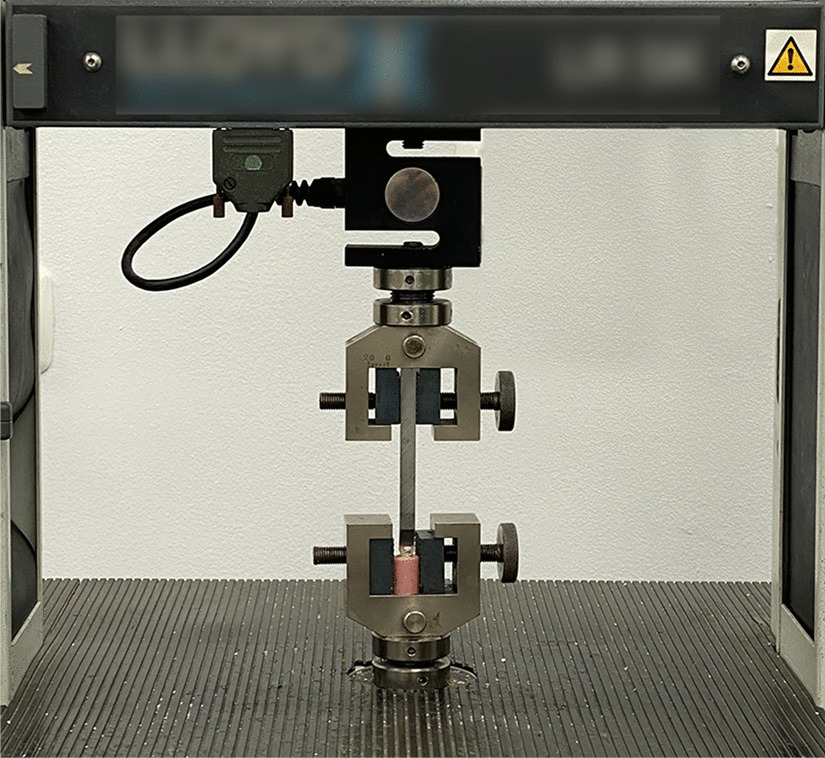
The mold was fixed firmly with the screw on a holding ring of the universal testing machine

**Fig. 3:**
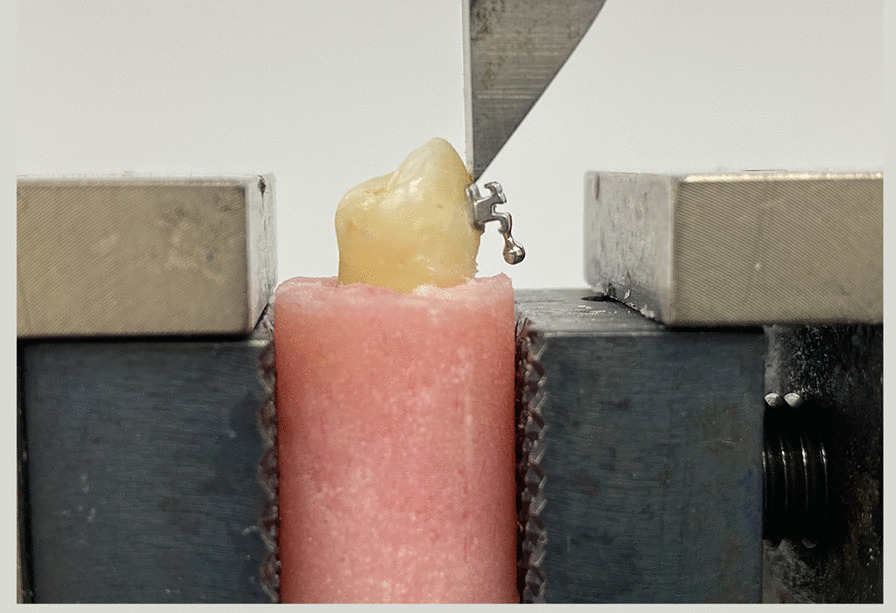
Force applied by the universal testing machine tapered blade between the bracket base and the tooth

The SBS was measured using a universal testing machine (LR 5 K Lloyd, UK) with the cross-head speed adjusted to 0.5 mm/min. Each mold was fixed on a holding ring and fixed firmly with the screws in the lower table of the universal testing machine (Fig. 2). The machine tapered blade applied force between the bracket base and the tooth (Fig. 3) and recorded the required force to debond each bracket in Kilograms on a monitor. The measurements were later converted to megapascals (MPa).

### Adhesive remnant index determination

The amount of adhesive remaining on the tooth surface after debonding was assessed using a stereomicroscope (Olympus SZ-CTV, Japan). One blinded examiner determined the scores through evaluation of the remaining adhesive on the enamel surface at 20X and 40X magnifications. Digital photographs of each tooth were recorded at each magnification. Each tooth was assigned an ARI score from 0 to 3 as described by Årtun and Bergland [33]:

- Score 0 was assigned when no adhesive remained on the tooth surface; indicating that the bond failure occurred entirely at the resin/enamel interface.
- Score 1 was assigned when less than half the adhesive remained on the tooth surface; indicating that the bond failure occurred predominantly at the resin/enamel interface.
- Score 2 was assigned when more than half of the adhesive remained on the tooth surface; indicating that the bond failure occurred predominantly at the bracket/resin interface.
- Score 3 was assigned when all adhesive remnants are on the tooth surface; indicating that the bond failure occurred entirely at the bracket/resin interface [40].

The scores calibration was repeated by the same examiner after 2 weeks for reliability. Calibration on ARI assessment was done and kappa statistic was calculated (K = 0.79) indicating very good intra-examiner reliability [36].

### Statistical analysis

Normality was checked for SBS using descriptive statistics, plots, and normality tests. Mean, standard deviation, 95% confidence interval and range were calculated for shear bond strength, while frequencies and percentages were calculated for Adhesive Remnant Index (ARI). Comparison between the three study groups was done using One-Way-ANOVA for shear bond strength, and Kruskal Wallis test for the ARI index. Significance was set at *p* value < 0.05. Data was analyzed using IBM SPSS for windows version 23.0.

## Results

**Fig. 4:**
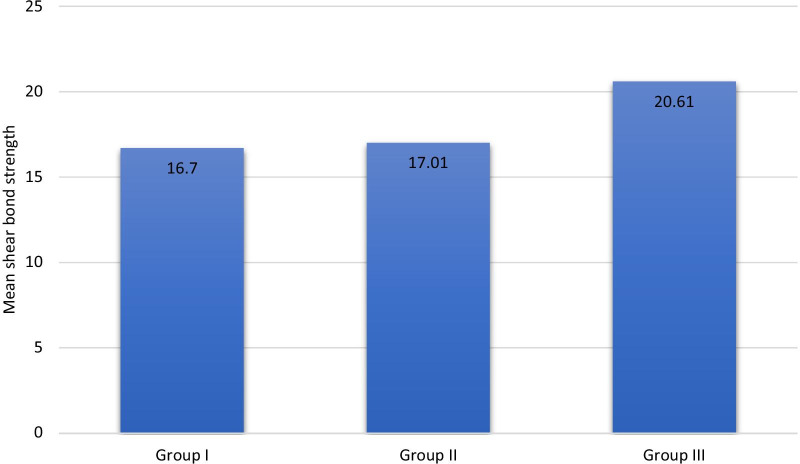
The mean SBS values among the three groups

Shear bond strength values showed normal distribution in the three groups (P > 0.05). In this study, the mean SBS values of the 3 experimental groups were 16.7 ± *5.63*, 17.01 ± *1.30* and 20.61 ± *7.88*, respectively. However, One-Way-ANOVA revealed no statistical significance difference between them (P = 0.17). Table 1 shows the descriptive statistics of shear bond strength. Figure 4 represents the mean SBS values among the three groups.

**Table 1:** Shear bond strength in the three study groups

	SBS mean ± SD	95% CI	Minimum–maximum
Group I	16.70 ± 5.63	14.07, 19.34	8.05–26.63
Group II	17.01 ± 1.30	13.49, 20.53	5.29–31.24
Group III	20.61 ± 7.88	16.81, 24.40	9.08–32.84
F of ANOVA P value	F = 1.86 P = 0.17

**Fig. 5:**
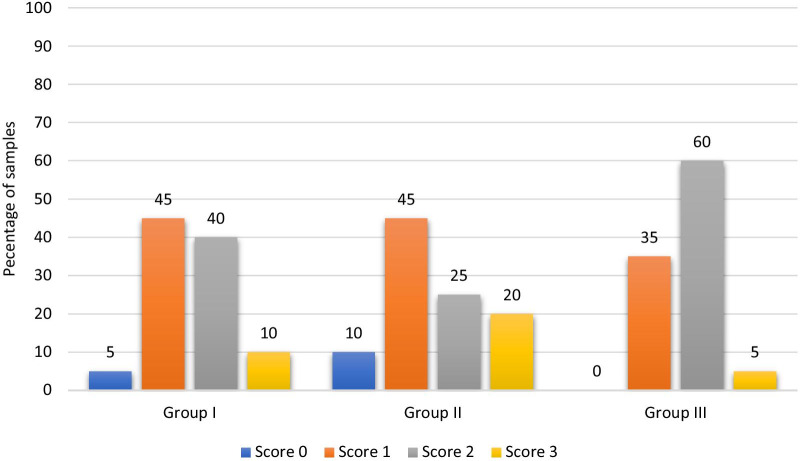
The ARI score percentages among the 3 groups

The ARI score percentages are reported in Table 2 as well as Fig. 5. Kruskal Wallis test showed no statistically significant difference between the three groups (P = 0.69).

**Table 2:** Adhesive Remnant Index (ARI) in the three study groups

	Group I	Group II	Group III	Kruskal–Wallis test P value
	N (%)			
Score 0	1 (5%)	2 (10%)	0 (0%)	Z = 0.75 P = 0.69
Score 1	9 (45%)	9 (45%)	7 (35%)	
Score 2	8 (40%)	5 (25%)	12 (60%)	
Score 3	2 (10%)	4 (20%)	1 (5%)	
Median (IQR)	1.50 (1.00)	1.00 (1.00)	2.00 (1.00)	

Z: Kruskal–Wallis test; IQR: Interquartile range.

## Discussion

Er,Cr:YSGG is one of the most used lasers in the attempt to decrease enamel demineralization [18]. Laser increases enamel resistance by modifying the morphology, chemical composition or solubility of enamel rather than ablating the enamel surface [37]. Sub-ablative laser parameters were used based on previous studies [24, 37].

Preservation of the sound enamel surface is important at the end of the orthodontic treatment [38]. However, choosing a preventive measure that will not negatively affect the SBS of the orthodontic bracket is a must to guarantee a successful treatment of about 2 years [39]. Ideally, the bonded brackets should be strong enough to withstand the orthodontic and masticatory forces without failure throughout the treatment period but should be debonded at the end of the treatment without causing any damage to the tooth structure [39]. The required bond strength of orthodontic brackets ranges between 5.9 and 7.8 MPa [40]. This could be challenging if the enamel surface is to be pretreated before bonding.

In the current study, the enamel surface was irradiated by sub-ablative Er,Cr:YSGG laser and the results showed no statistically significant difference between the no pretreatment group (control) and the lased group [39]. This agrees with Roshan and Hosseini [41] as well as Lopes et al. [42] who evaluated the SBS after Er,Cr:YSGG laser etching and found no statistically significant difference in the SBS of orthodontic brackets bonded to enamel surface after acid etching (control) and Er,Cr:YSGG laser conditioning. On the other hand, Mollabashi et al. [43] found a statistically significant reduction in the SBS of metallic brackets after Er,Cr:YSGG laser etching but the bond strength was clinically acceptable. Only Baglar [15] evaluated the SBS after applying sub-ablative Er,Cr:YSGG as a preventive aid before ceramic veneer restorations, concluding that Er,Cr:YSGG laser pretreatment did not have a negative effect on the shear bond strength.

The use of CPP–ACP as a preventive measure showed promising results compared to fluoride [13], but its effect on shear bond strength of orthodontic brackets is controversial. Naseh et al. [44] Cossellu et al. [45] Ladhe [46] Lu et al. [47] Veli et al. [48] and Park et al. [49] evaluated the SBS after CPP-ACP pretreatment on both sound or bleached and demineralized enamel and found no statistically significant difference between the CPP-ACP pretreated enamel and the control groups. Although Ladhe et al. [46] noted a significant reduction in the SBS when chemically cured composite was used, such reduction was clinically acceptable. On the other hand, Cehreli et al. [27] found a significant and clinically unacceptable decrease in the SBS when CPP-ACP was applied before acid etch. Nonetheless, Khargekar et al. [25] revealed a significant increase in the SBS after CPP-ACP pretreatment when compared to fluoride pretreatment and no pretreatment groups.

Combining Er,Cr:YSGG laser with CPP-ACP is a recent attempt to control enamel demineralization. This combination showed a significant decrease in WSLs’ depth compared to the control group [24]. However, to our knowledge, no previous studies evaluated the SBS of metallic brackets after using sub-ablative Er,Cr:YSGG combined with the CPP-ACP. The results of this study revealed no significant difference in shear bond strength after using Er,Cr:YSGG combined with the CPP-ACP. This suggests that such a combination could be used before bonding orthodontic brackets.

Adhesive remnant index is one of the most frequently used indices that evaluate the amount of remaining adhesive on the enamel surface after bracket debonding [35]. The efficiency of the ARI to reflect the bond strength is debatable [27, 44, 45, 48, 50–52]. However, The index determines the bond failure site after assigning each tooth a score from 0 to 3. The less adhesive remaining on the enamel after the debonding procedure, the safer the enamel clean up [45, 53], hence the less the risk of enamel damage. Nonetheless, the presence of some composite remaining at the enamel surface may indicate less risk of enamel fracture during bracket removal [50, 54]. In this study, no statistically significant differences were found regarding the ARI scores between the 3 groups where most of the scores were either 1 or 2 in all the groups, indicating a cohesive failure [34]. Hence, the same amount of enamel surface protection is established with or without applying the preventive measure, as the potential risk of enamel fracture during debonding and enamel damage during enamel clean-up after debonding is minimized.

This study was conducted as an in-vitro study to have a more standardized bonding protocol allowing independent evaluation of the SBS of orthodontic brackets [39]. Replication of the oral environment was done by utilizing extracted human premolars teeth rather than bovine teeth that have shown to dissolve two or three times faster than human enamel [55], storing teeth in artificial saliva and subjecting teeth to thermocycling that is equivalent to one year in the oral environment [32, 56]. Nevertheless, it is nearly impossible to replicate all the oral environment factors in in-vitro studies and this is the limitation of our study. It is recommended to conduct in-vivo studies to test the failure rate of orthodontic brackets bonded after Er,Cr:YSGG Laser and CPP–ACP application.

## Conclusions

- The SBS of metallic brackets bonded to enamel surface pretreated with sub-ablative Er,Cr:YSGG laser was comparable to those bonded to non-pretreated enamel.
- Enamel pretreatment using Er,Cr:YSGG combined with the CPP-ACP before bonding orthodontic brackets, does not endanger the shear bond strength.
- Applying Er,Cr:YSGG alone or combined with CPP-ACP showed no effect on ARI scores (nor failure mode) upon bracket debonding.

## Data Availability

The datasets used during the current study are available from the corresponding author on reasonable request. All data analyzed during this study are included in this published article in the form of tables and figures.

## Abbreviations

WSLs: White spot lesions
CPP-ACP: Casein phosphopeptide amorphous calcium phosphate
NdYAG: Neodymium-doped yttrium aluminum garnet
ErYAG: Erbium-doped yttrium aluminum garnet
Er,Cr:YSGG: Erbium, chromium: yattrium-scandium-gallium-garnet
CPP-ACPF: CPP-ACP with fluoride
SBS: Shear bond strength
ARI: Adhesive remnant index
MPa: Megapascals
